# M-protein diagnostics in multiple myeloma patients using ultra-sensitive targeted mass spectrometry and an off-the-shelf calibrator

**DOI:** 10.1515/cclm-2023-0781

**Published:** 2023-10-12

**Authors:** Charissa Wijnands, Pieter Langerhorst, Somayya Noori, Jenneke Keizer-Garritsen, Hans J.C.T. Wessels, Jolein Gloerich, Vincent Bonifay, Hélène Caillon, Theo M. Luider, Alain J. van Gool, Thomas Dejoie, Martijn M. VanDuijn, Joannes F.M. Jacobs

**Affiliations:** Department of Laboratory Medicine, Radboud University Medical Center, Nijmegen, The Netherlands; Department of Neurology, Erasmus MC, University Medical Center Rotterdam, Rotterdam, The Netherlands; Sebia, Lisses, France; Biochemistry Laboratory, Hospital of Nantes, Nantes, France

**Keywords:** multiple myeloma, targeted mass spectrometry, clonotypic peptide, minimal residual disease, M-protein

## Abstract

**Objectives:**

Minimal residual disease status in multiple myeloma is an important prognostic biomarker. Recently, personalized blood-based targeted mass spectrometry (MS-MRD) was shown to provide a sensitive and minimally invasive alternative to measure minimal residual disease. However, quantification of MS-MRD requires a unique calibrator for each patient. The use of patient-specific stable isotope labelled (SIL) peptides is relatively costly and time-consuming, thus hindering clinical implementation. Here, we introduce a simplification of MS-MRD by using an off-the-shelf calibrator.

**Methods:**

SILuMAB-based MS-MRD was performed by spiking a monoclonal stable isotope labeled IgG, SILuMAB-K1, in the patient serum. The abundance of both M-protein-specific peptides and SILuMAB-specific peptides were monitored by mass spectrometry. The relative ratio between M-protein peptides and SILuMAB peptides allowed for M-protein quantification. We assessed linearity, sensitivity and reproducibility of SILuMAB-based MS-MRD in longitudinally collected sera from the IFM-2009 clinical trial.

**Results:**

A linear dynamic range was achieved of over 5 log scales, allowing for M-protein quantification down to 0.001 g/L. The inter-assay CV of SILuMAB-based MS-MRD was on average 11 %. Excellent concordance between SIL- and SILuMAB-based MS-MRD was shown (R^2^>0.985). Additionally, signal intensity of spiked SILuMAB can be used for quality control purpose to assess system performance and incomplete SILuMAB digestion can be used as quality control for sample preparation.

**Conclusions:**

Compared to SIL peptides, SILuMAB-based MS-MRD improves the reproducibility, turn-around-times and cost-efficacy of MS-MRD without diminishing its sensitivity and specificity. Furthermore, SILuMAB can be used as a MS-MRD quality control tool to monitor sample preparation efficacy and assay performance.

## Introduction

Multiple myeloma (MM) is the second most common hematologic malignancy that is characterized by the clonal expansion of plasma cells in the bone marrow and production of a monoclonal immunoglobulin (M-protein) [1]. Although MM is incurable, therapeutic strategies have improved rapidly over the past years, leading to an increased percentage of newly diagnosed MM patients who reach a stringent complete remission [2]. This has led to improved efforts to measure minimal residual disease (MRD) which is defined as one myeloma cell in ≥10^5^ nucleated cells by the International Myeloma Working Group [3]. Bone marrow-based MRD assays are sensitive, but performing biopsies is invasive. The M-protein provides a biomarker that is detected in peripheral blood of MM patients. The current gold standard for M-protein diagnostics is serum protein electrophoresis (SPEP) to quantify circulating M-protein and immunofixation electrophoresis to identify M-protein isotype combined with immunoassays to quantify serum free light chains [4]. Alternatively, in 2018 the Mayo clinic successfully implemented high throughput intact protein mass spectrometry to detect M-proteins (MASS-FIX) to replace immunofixation electrophoresis in routine clinical care [5]. In addition, MASS-FIX is able to identify novel M-protein features such as light chain glycosylation and it is less prone to be affected by therapeutic monoclonal antibody therapy [6], [7], [8]. Although cost-effective and easy in use, the sensitivity of all the above-mentioned blood tests is insufficient to measure MRD. Therefore, there is a clinical need for more sensitive methods to monitor M-proteins.

One promising method is liquid chromatography – tandem mass spectrometry (LC-MS/MS) for MRD measurements in MM patient blood (MS-MRD). MS-MRD is achieved by quantitating unique clonotypic peptides derived from the variable region of the M-protein by enzymatic digestion followed by LC-MS/MS [9], [10], [11]. The selected clonotypic peptides act as surrogate biomarkers for the M-protein and arise through *V*(*D*)*J*-gene rearrangements and somatic hypermutations in both the heavy- and light chain variable region [12, 13]. Recent studies by us and others demonstrated that MS-MRD is 1,000-fold more sensitive compared to SPEP and can be used to monitor MRD patients [9], [10], [11, 14, 15].

Quantification of the M-protein is an important step in MS-MRD workflows and most MS-MRD studies have used stable isotope labelled (SIL) peptides as internal calibrator [11, 14, 16]. SIL peptides have proven to be a powerful tool in targeted proteomics for the quantification of proteins [17]. However, the use of SIL peptides also presents challenges. For each patient, unique SIL peptides must be synthesized and evaluated, which is a time-consuming and costly process. Furthermore, harmonization between laboratories is difficult because (clonotypic) peptide detectability can be LC-MS/MS system dependent [18]. Therefore, a universal calibrator that provides diverse peptides to quantify patient specific clonotypic peptides would aid the implementation of MS-MRD in clinical practice to monitor M-proteins in blood of MM patients with MRD.

Previous research by McDonald et al. and Liyasova et al. showed the feasibility to normalize M-protein-derived peak areas to calculate relative M-protein levels [9, 19]. In their presented research, a single M-protein target is selected from the M-protein sequence obtained by *de novo* protein sequencing, and an artificial protein (Digestif) is used for normalization. While, this work demonstrates an important step towards a more generic MS-MRD assay, the calibrator of choice is an artificial protein that differs in nature compared to the M-protein analyte. Another limitation is the use of a single M-protein-derived peptide which does not cover both the heavy chain and light chain of the M-protein.

Here, we introduce an improved adaption of the personalized MS-MRD assay by applying a commercially available stable-isotope labelled human IgG-Kappa (SILu™MAB K1, SILuMAB) as an off-the-shelf calibrator. We show that SILuMAB-based MS-MRD allows for accurate and reproducible M-protein quantification over five orders of magnitude by making use of multiple M-protein-derived peptides. In addition to its quantification potential, we assessed the application of SILuMAB as quality control (QC) on various aspects of the MS-MRD assay. We conclude that incorporation of SILuMAB into the MS-MRD blood-test provides a universal platform for patient specific M-protein quantification by LC-MS/MS, which greatly aids the implementation of MS-MRD in clinical practice.

## Materials and methods

A total of 284 sera from 13 patients were collected from the IFM-2009 clinical trial (ClinicalTrials.gov identifier NCT01191060) [20]. Patient characteristics are described in Supplementary Table S1. Written informed consent and clinical and genomic data were de-identified in accordance with the Declaration of Helsinki, and approval for this study was provided by our Institutional Review Board (2018-4140).

The M-protein-specific clonotypic peptide targets were selected based on bioinformatic analysis of a 100,000-read sample extracted from RNA sequencing data as previously described [14, 21]. Protein digestion was performed as reported previously [16] and a more detailed description of the methods and reagents can be found in the supplementary methods. Peptides were separated on a liquid chromatography system (Evosep one). On C18 Evotips (Evosep), 125 ng of digested serum was loaded as per manufacturer’s instructions. The eluted peptides were analyzed on the timsTOF Pro 2 (Bruker Daltonics, Bremen, Germany) operated in Parallel accumulation-serial fragmentation (PASEF) mode. For quantification of the peptides, PRM-PASEF was used. Here, peptide identification results were used by Skyline software to generate a PRM-PASEF method using 5 min retention time windows. Clonotypic peptide targets were measured with optimized collision energies. The clonotypic peptides used for quantification are listed in Supplementary Table S3. Methods used by the Erasmus MC were described previously [16].

Data was analyzed as previously reported [14]. Subsequently, the peak areas were exported, and the M-protein concentrations were determined for each sample using the following formula:

$$\left[{M\;\text{protein}}_{\text{sample}}\right]=\frac{\left(\frac{\text{Clonotypic}\;\text{peak}\;\text{area}\;\text{in}\;\text{sample}}{\text{Average}\;\text{Silumab}\;\text{peak}\;\text{area}\;\text{in}\;\text{sample}}\right)}{\left(\frac{\text{Clonotypic}\;\text{peak}\;\text{area}\;\text{at}\;\text{intake}}{\text{Average}\;\text{silumab}\;\text{peak}\;\text{area}\;\text{at}\;\text{intake}}\right)}*\left[{M\;\text{protein}}_{\text{intake}}\right]$$

The M-protein concentration at screening was determined by SPEP. The formula was applied to each clonotypic peptide individually with average SILuMAB peptide intensities for each sample. The concentrations for each clonotypic peptide within a sample were averaged to report one final M-protein concentration. A more detailed description of the used methods can be found in the supplemental files.

## Results

### SILuMAB peptide and concentration selection

**Figure 1:**
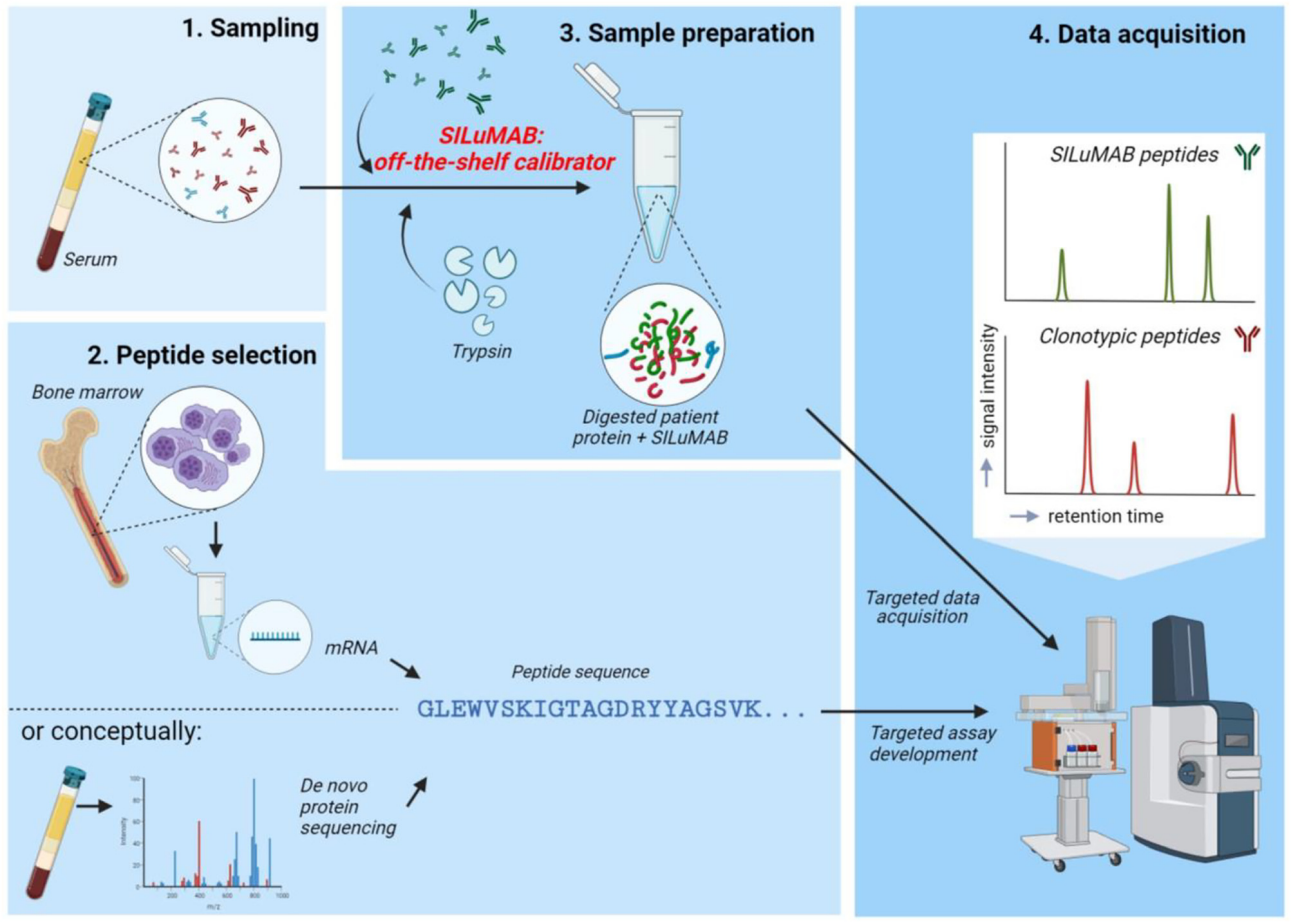
Workflow of SILuMAB-based MS-MRD. (1) Sampling: A patient serum sample is required. (2) Peptide selection: clonotypic peptides can be determined from M-protein mRNA obtained from bone marrow as implemented in this research. Alternatively, *de novo* sequencing can be used to determine the M-protein peptide sequence [9, 19, 22]. (3) Sample preparation: SILuMAB is spiked in the serum and the sample is digested. (4) Data acquisition: samples are analyzed by LC-MS/MS where data is acquired from both clonotypic and SILuMAB peptides. LC-MS/MS: liquid-chromatography tandem mass spectrometry. Figure was created with BioRender.com.

Quantification of MS-MRD data with SILuMAB offers a personalized M-protein monitoring method using an off-the-shelf calibrator (Figure 1). We characterized the SILuMAB derived tryptic peptides that could be reproducibly detected by LC-MS/MS since it is imperative for this method that suitable SILuMAB peptides are selected. This experiment was performed in two independent laboratories and minimal differences in peptide detectability and stability were observed (data not shown). Out of the ±20 detectable peptides, both laboratories using either timsTOF or orbitrap MS selected their top 5 SILuMAB peptides based on signal intensity, inclusion of heavy and light chain derived peptides, and coverage of a wide retention timespan (Supplementary Table S2; MS characteristics in Supplementary Table S3). We determined the lowest SILuMAB concentration that provides a robust and reproducible signal to minimize the amount of SILuMAB and maximize the amount of patient serum per injection. To this end, we serially diluted SILuMAB in control serum (ranging from 0.04 ng/μL to 10.7 ng/μL). Based on reproducibility and peptide detectability, 1 ng/μL was selected as the optimal spike concentration (data not shown). This corresponds to approximately 1 % of the total protein concentration in the digested serum.

### Validation of the SILuMAB-based MS-MRD assay

#### Sensitivity and dynamic range of the SILuMAB-based MS-MRD assay

**Figure 2:**
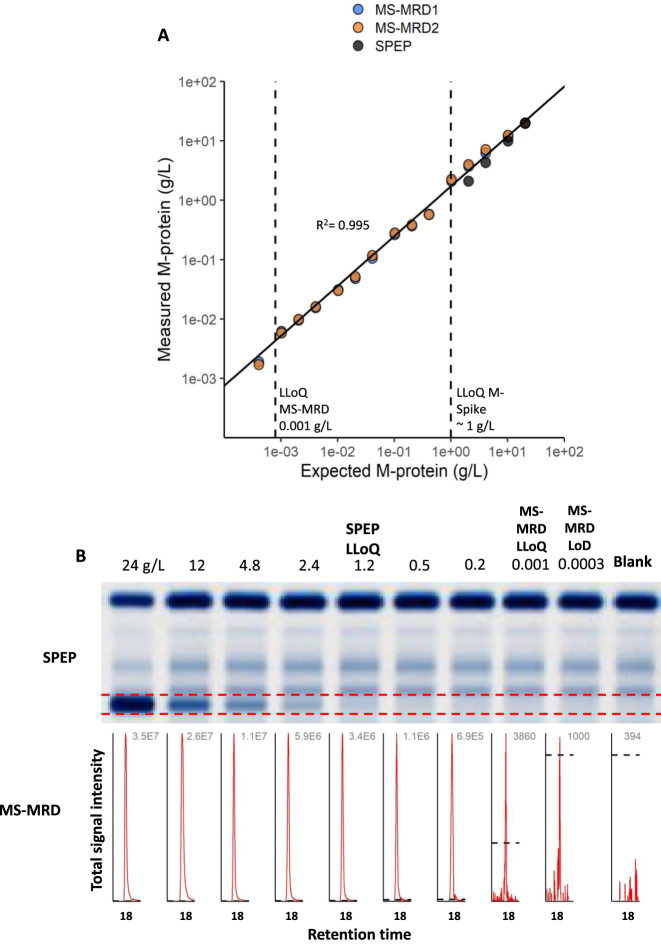
Linearity and dynamic range of SILuMAB-based MS-MRD. (A) Serum with 24 g/L IgA-Lambda M-protein was serially diluted in control serum. (B) The same diluted samples were analyzed with SPEP. The corresponding MS-MRD signals of a representative clonotypic peptide (AIGPVISR) are shown in grey with the signal of the LoD indicated in each peak by the black dotted line. LoD, limit of detection; LLoQ, lower limit of quantification.

Serum containing 24 g/L IgA-Lambda M-protein was serially diluted in control serum down to 0.0001 g/L and quantified using the SILuMAB-based MS-MRD workflow to assess the dynamic range and sensitivity of MS-MRD with SILuMAB as a calibrator. We observed a linear signal (R^2^=0.996) over the range of five log scales (Figure 2A), a LoD (limit of detection) of 0.0007 g/L, and a LLoQ (lower limit of quantification) of 0.001 g/L. Additionally, the diluted samples were analyzed using SPEP (Figure 2B) showing a quantifiable monoclonal band down to 1.2 g/L. This experiment was repeated on serum from patient 2 which contained 64 g/L monoclonal IgG Kappa (Supplementary Table S4A). We observed an R^2^ of 0.994, a LoD of 0.001 g/L and a LLoQ of 0.002 g/L. SPEP analysis of the same diluted samples from patient 1 showed a quantifiable monoclonal signal down to 3 g/L, but traces were detectable down to 1 g/L. These results are in line with the generally accepted LoD and LLoQ of SPEP [23, 24].

Overall, we observed a 1,000-fold improvement in sensitivity of SILuMAB-based MS-MRD compared to SPEP which is concordant with previously reported results of SIL-based MS-MRD [11].

#### Precision and reproducibility of the SILuMAB-based MS-MRD assay

**Figure 3:**
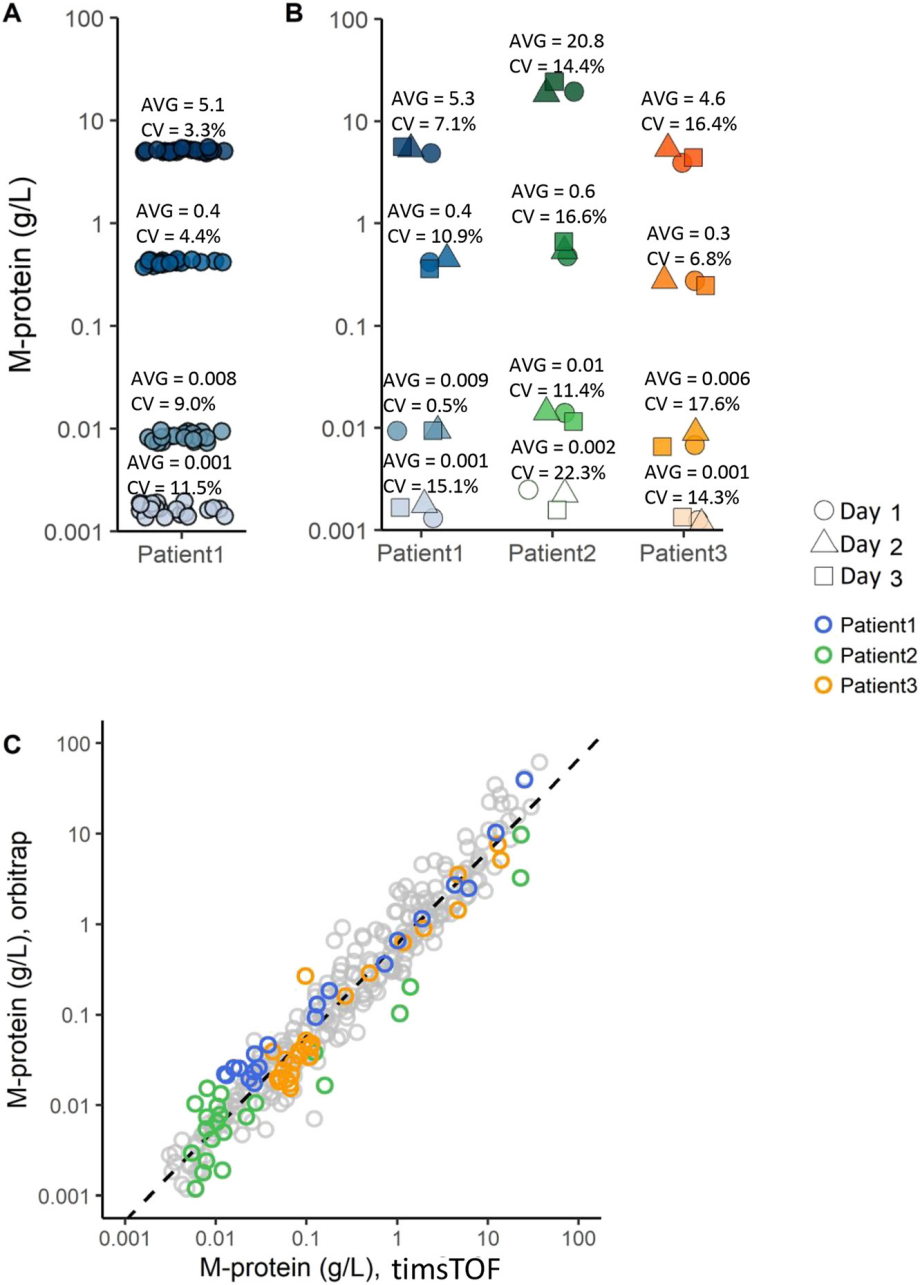
Reproducibility of SILuMAB-based MS-MRD. (A) Intra-assay variation. Twenty measurements of four sera from patient 1 containing high, medium, low and LLoQ M-protein concentrations. (B) Inter-assay variation. Each shape represents one day measurements. Average and %CV calculated over three days. (C) Between-laboratory variation. 284 samples of 13 patients measured at two different laboratories. Patients 1, 2 and 3 are highlighted. LLoQ, lower limit of quantification.

Generating precise and reproducible results is indispensable for reliable monitoring of M-protein concentrations over time. To this end, we assessed the precision and inter-laboratory variation of SILuMAB-based MS-MRD. To assess the precision, an intra- and inter-assay variation analysis were performed. The intra-assay variation was determined by preparing three samples with a high, medium, low and LLoQ M-protein concentration of patient 1. All samples were measured 20 consecutive times which resulted in an intra-assay CV ≤9 % for the high, medium and low M-protein concentration and 11.5 % for the LLoQ M-protein concentration (Figure 3A). Inter-assay variation was estimated by three individual digests of a high, medium, low and LLoQ sample from patient 1. Each individual digest was prepared and analyzed on a separate day and measured five times each as recommended by the FDA [25]. This experiment was repeated on samples from two additional patients and all resulting CVs of the high, medium and low M-protein concentrations were ≤±15 % and ≤±20 % at LLoQ concentration (Figure 3B). This indicated a technically reproducible method that is in line with the FDA acceptance criteria. To determine the variation of SILuMAB-based MS-MRD between two different laboratories, 284 longitudinally sampled sera obtained from 13 patients were prepared and acquired by two different laboratories using different LC-MS/MS platforms (timsTOF and orbitrap). Results in Figure 3C, showed an R^2^ of 0.939 and slope of 1.02, indicating sufficient agreement between both laboratories. However, because of inequivalent absolute quantification, the MS-MRD methods cannot be used interchangeably between laboratories in case a patient is transferred to a different hospital. Overall, the reproducibility experiments indicate good precision and reproducibility of the SILuMAB-based MS-MRD assay.

#### SILuMAB-based MS-MRD compared to SIL peptide-based MS-MRD

**Figure 4:**
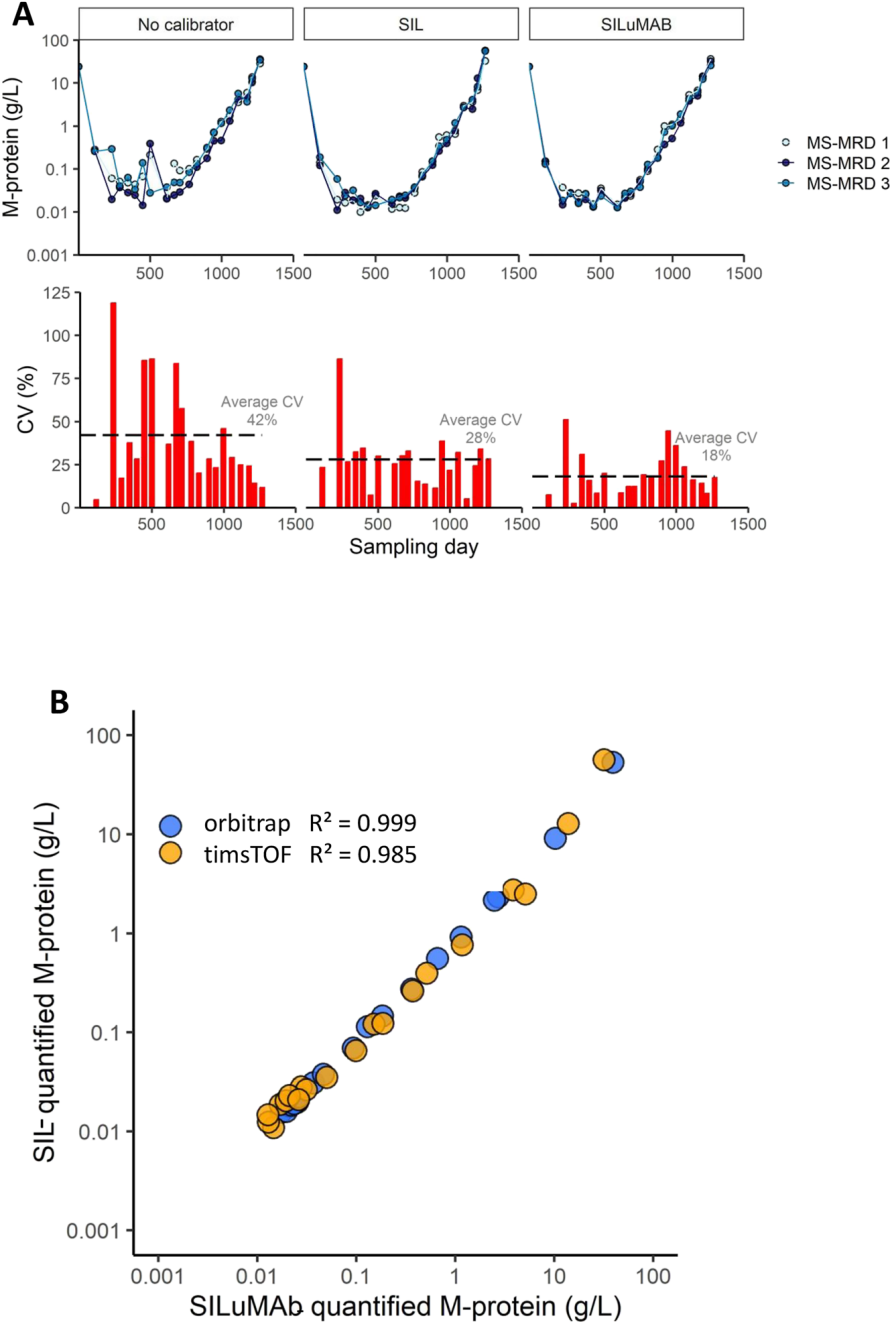
Comparison of SIL- and SILuMAB quantified MS-MRD data. (A) Stability demonstrated on three independent triplicates (MS-MRD1-3) of sera from patient 1. No calibrator (left), SIL (middle), and SILuMAB (right). Upper graphs show M-protein concentrations; lower graphs show %CVs of the three measurements per sample. (B) Samples from patient 1 quantified with SIL or SILuMAB by different laboratories. MS-MRD, mass spectrometry minimal residual disease; SIL, stable isotope labeled peptides.

To test the beneficial effect of SILuMAB over MS-MRD quantification based on label free quantification (LFQ) or SIL peptides, 21 longitudinally collected sera from patient 1 were prepared with both SIL peptides and SILuMAB. We investigated the reproducibility of three M-protein quantification methods in MS-MRD: without calibrator (TIC (total ion current) normalization was applied), SIL peptide-based and SILuMAB-based (Figure 4A). This experiment was performed in independent triplicates. The highest CVs were observed when no calibrator was used for quantification (average CV=42 %). However, the reproducibility drastically improved with the use of either SIL peptides (average CV=28 %) or SILuMAB (average CV=18 %). The lowest CVs were observed when SILuMAB-based MS-MRD was employed. A major difference between SIL-based and SILuMAB-based MS-MRD is the use of a single M-protein-derived peptide compared to the average of multiple M-protein derived peptides. A significantly improved CV (18 %) was observed when calculating the average M-protein concentration over three M-protein-derived peptides compared to the results of any single M-protein-derived peptide (25–39 %) (Supplementary Figure S5). This experiment was repeated on longitudinally collected sera from two additional patients and similar results were observed (Supplementary Table S4B). These data show that a calibrator to correct for technical and experimental variation in MS-MRD data is indispensable for reproducible and reliable results. The use of SILuMAB has shown to provide superior reproducibility and reliability compared to other methods to this end.

Previously, we showed adequate linearity, sensitivity and accuracy of SIL peptide based MS-MRD using SIL peptides for M-protein quantification [11]. Furthermore, MS-MRD allowed for longitudinal monitoring of MM patients [16, 22, 26]. To assess the concordance between MS-MRD data quantified by SIL peptides and SILuMAB, all available longitudinal samples from three patients were digested with both SILuMAB and SIL peptides (data for patient 1 is shown in Figure 4B and other data is available in Supplementary Table S4C). This experiment was performed at two laboratories using different LC-MS/MS systems (timsTOF and orbitrap). For both laboratories and for all three patients, R^2^s of >0.985 were observed, indicating an excellent agreement between MS-MRD M-protein quantification using SIL-peptides or SILuMAB.

### SILuMAB as quality control (QC) for MS-MRD

LC-MS/MS system performance and high quality repeatable digestions are essential for accurate and reliable MS-MRD measurements. We assessed whether SILuMAB can also function as a QC for analytical workflow performance and digestion-efficacy of individual samples in the MS-MRD assay.

**Figure 5:**
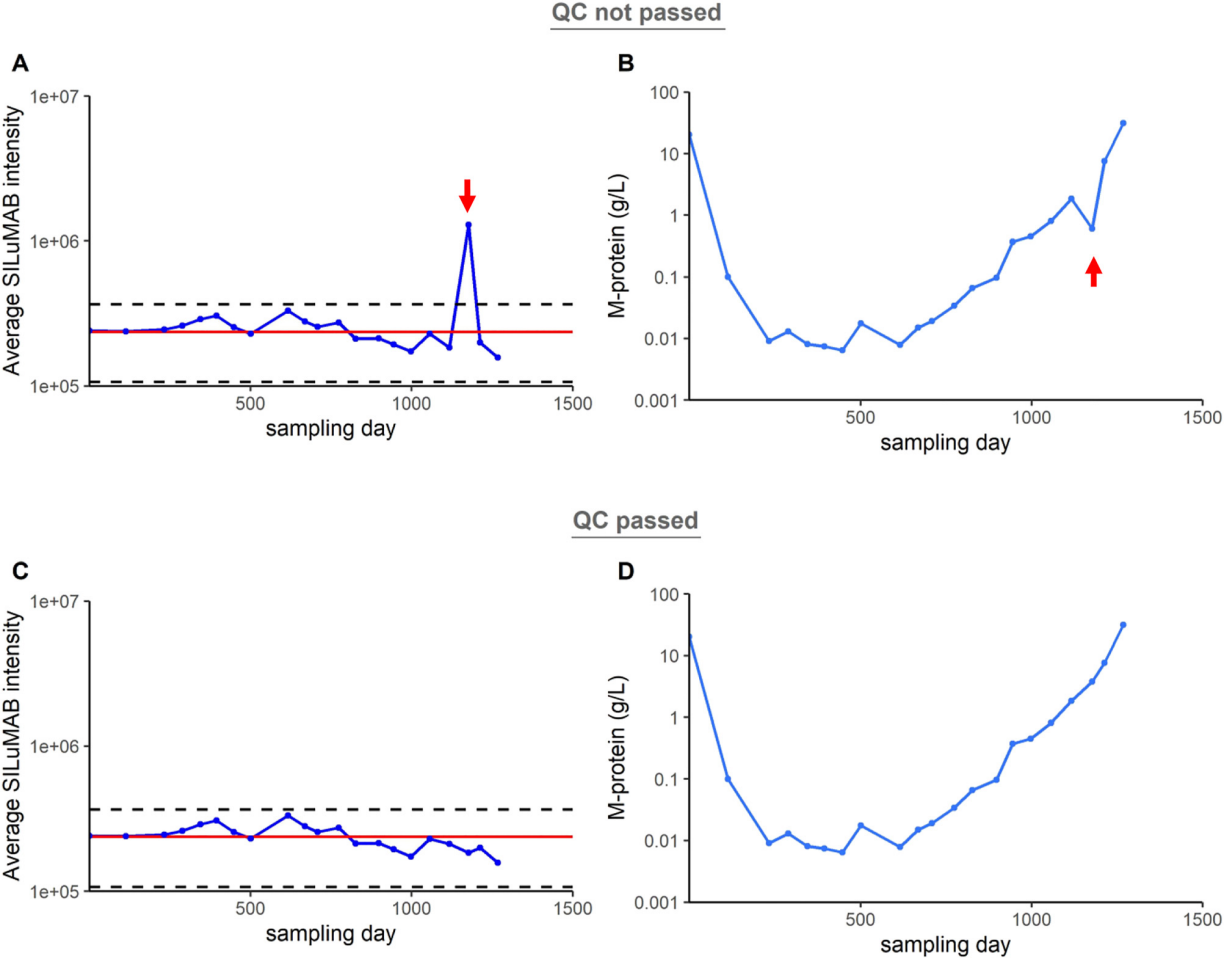
SILuMAB signal intensity as QC tool for MS-MRD. (A) Average SILuMAB signal in sera from patient 1 (blue line) and ±3SD from SILuMAB average over all samples (black dotted lines). One sample (red arrow) did not pass the criteria. (B) Resulting in an underestimation of the M-protein. (C) All samples in the series met the criteria after re-digestion of this sample and (D) correct M-protein concentrations were observed.

The variation in SILuMAB peptide signal intensity between measured samples can be used to monitor run specific system performance and may flag suboptimal analytical workflow performance. In addition to the criterium that the CV of the average signal intensity calculated over all samples in a series should be <20 %, we used the standard deviation of the average SILuMAB signal intensity over a series of measurement to create outer limits (average ± 3*SD). If the SILuMAB signal for a specific sample surpasses these limits this could indicate a sample specific problem, and warrants re-analysis or re-digestion of the sample. Figure 5A shows an example of a series of measurements of which one data point, indicated by the red arrow, did not meet the QC criteria. This resulted in an underestimation of the M-protein concentration (Figure 5B). After re-digestion and re-analysis, all samples met the QC criteria (Figure 5C) and no unexpected decrease in M-protein concentration was observed (Figure 5D).

**Figure 6:**
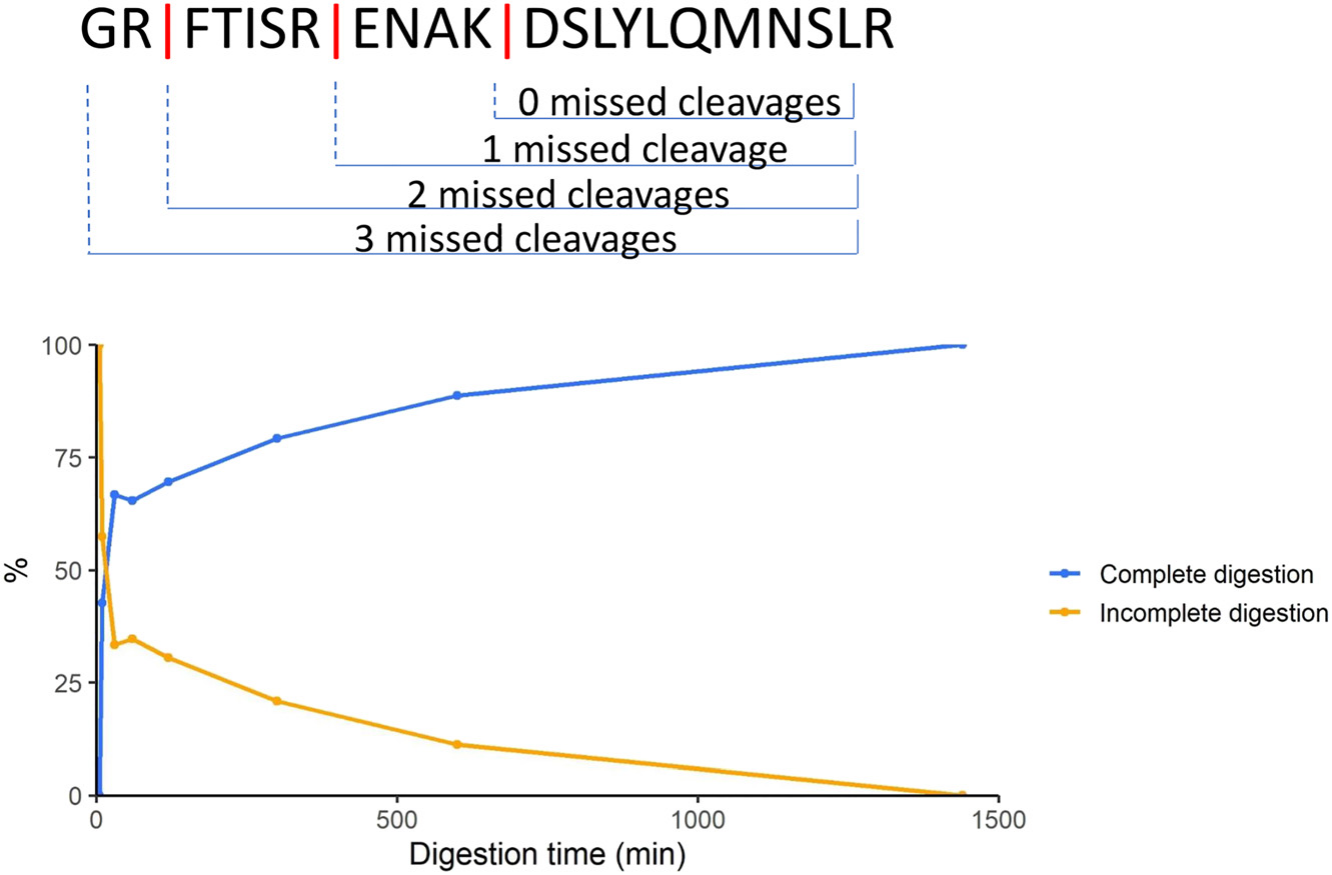
SILuMAB missed cleavages as QC tool for MS-MRD. The fully cleaved SILuMAB peptide (DSLYLQMNSLR) and its missed cleaved products are depicted (top panel). The bottom panel displays the relative intensity (normalized to the total observed intensity) of the fully cleaved (blue line) and missed cleaved peptides (orange line) over the course of 5–1440 min digestion.

Incomplete protein digestion results in decreased sensitivity and non-reproducible signal intensities in the MS-MRD assay. Therefore we explored the possibility of using SILuMAB as a QC for digestion efficiency. In general, complete digestion is reached during an overnight incubation [27]. We simulated incomplete digestion by digesting SILuMAB with various digestion times (ranging from 5 to 1,440 min). Subsequent LC-MS/MS analysis and database analysis were performed to identify peptides with missed cleavages, which arise during incomplete digestion. Peptide DSLYLQMNSLR and all of its missed cleaved products were identified to evaluate the digestion efficiency (Figure 5B). As expected, a digestion time of 5 min resulted in 100 % products with at least one missed cleavage site (orange line in Figure 6) corresponding to incomplete digestion. The correct and fully digested peptide increased in intensity with longer digestion times and the missed cleaved peptides decreased in intensity down to 0 % at 24 h digestion. By monitoring specific SILuMAB peptides and their missed cleaved products, the digestion efficiency can be monitored sample wise. These findings highlight that next to MS-MRD quantification, SILuMAB can also function as QC quantifier in MS-MRD.

## Discussion

M-protein diagnostics play a key role in monitoring patients with MM. Improvements in MM treatment increased the number of patients who reach a state of minimal residual disease. Beyond stringent complete remission, disease load cannot be monitored sufficiently by routine gel-based M-protein diagnostics. In recent years, we and others have shown that M-protein monitoring by MS-based techniques greatly improves sensitivity, and enables MRD monitoring in the blood of patients with MM. This is achieved by monitoring unique clonotypic M-protein peptides by LC-MS/MS. These personalized assays make use of patient-specific calibrator peptides. However, the synthesis of SIL peptides is costly and complicates harmonization between laboratories. Moreover, SIL peptide instability during storage may hamper accurate M-protein quantification. In this study, we introduce personalized MS-MRD M-protein quantification with a generic heavy labeled immunoglobulin calibrator (SILuMAB) that is commercially available as high-quality MS certified standard.

The advantage of using a SIL protein (SILuMAB) rather than SIL peptides, next to increased robustness, is the ease of harmonization of MS-MRD analyses on different LC-MS/MS systems. In this study we showed that two independent laboratories using different LC-MS/MS systems, and different SILMAB peptides for MS-MRD quantification obtained highly comparable results. Peptide detectability relies on the LC-MS/MS system used, as well as sample preparation methods employed [18, 28]. In practice, this means that clonotypic peptides can display different sensitivity and robustness in LC-MS/MS systems of various vendors. Using SILuMAB, each laboratory can select their own most optimal performing clonotypic peptides and still use the same universal calibrator.

The quantification method is based on the relative ratio between clonotypic peptides and SILuMAB derived peptides in sera with known M-protein concentration that can be used in the MRD samples. A limitation of the use of SILuMAB is the requirement of the availability of serum with a known M-protein concentration. We used the screening sample, but any patient sample with an M-protein of >3 g/L measured with SPEP can be used to quantify other samples of that particular patient. This threshold is based on previous research showing an inaccurate M-protein quantification when M-protein titers fall below 3 g/L using SPEP [29]. In clinical trials such samples are stored routinely and available for the majority of patients.

We showed that SILuMAB-based MS-MRD provided an excellent linear M-protein quantification over five log scales, with LLoQ between 0.002 and 0.001 g/L and LoD between 0.001 and 0.0003 g/L. The amount of added SILuMAB is relatively low (<1 % of total analyzed protein content) and data were concordant with the SIL-quantified MS-MRD data. The lower limits of SILuMAB-based MS-MRD are in line with previously described clonotypic peptide assays, in which a quantification limit was found to be approximately 0.001 g/L [9, 11, 14]. We showed that SILuMAB-based MS-MRD allows dynamic M-protein monitoring far beyond stringent complete remission and may be used for early relapse detection as such.

Normalization efforts to generate relative M-protein levels were previously introduced by Martins et al. and Liyasova et al. [9, 10]. In this research we observed values for LoD, LLoQ, and linearity that are similar to the results reported previously [9, 19]. However, in this validation process we also included intra-, inter-assay, and between-laboratory variation testing [19]. Finally, for the first time, we can now relate relative quantification to absolute M-protein quantification by the addition of SILuMAB, which improves harmonization with SPEP and eases the implementation of MS-based techniques in clinical practice. SPEP-based M-protein quantification has a reported inter-assay CV of approximately 15 % for M-proteins >3 g/L [29]. In this study we reported an average inter-assay CV of 11.3 % for SILuMAB-based MS-MRD M-protein quantification calculated in samples ranging from 0.001 to 24 g/L. This CV falls within the acceptance criteria of the FDA (CV≤±15 %; CV≤±20 % at LLoQ concentrations), indicating fit-for-purpose precision and reproducibility for SILuMAB-based MS-MRD [25]. Inter-laboratory variation testing showed a linear trend (R^2^=0.939) between the results from two different laboratories over 284 samples drawn from 13 patients which indicates sufficient relative agreement between both laboratories. However, we did observe considerable variations in absolute M-protein concentrations measured by both laboratories. Therefore, the feasibility of between-laboratory application should be further assessed before clinical use.

By comparing MS-MRD quantified by SIL and SILuMAB, we observed an improved reproducibility when using SILuMAB over SIL peptides. While these results seem counterintuitive, we observed that using SILuMAB to quantify multiple M-protein-derived peptides leads to a more stable assay performance compared to the analysis of a single M-protein target. Bias induced by single peptide analysis is corrected by averaging the resulting M-protein concentrations from multiple peptides.

Besides the use of SILuMAB in M-protein quantitation strategies, we also explored the use of SILuMAB as a QC parameter. We showed that SILuMAB can function as a sample specific digestion monitoring tool, based on the monitoring of incompletely digested peptides derived from SILuMAB. We showed that the variation in SILuMAB intensity can be used to monitor analytical MS-MRD workflow performance both batch- and sample-wise. The use of SILuMAB as QC for MS-MRD should be explored further to establish thresholds and guidelines for its use in clinical practice. Preliminary data show that the sensitivity of MS-MRD can be further improved by pre-analytical enrichment of immunoglobulins from serum [10, 14, 15]. Since SILuMAB is a human immunoglobulin, SILuMAB can be used as a QC tool for recovery efficiency of these pre-analytical sample preparation steps [10]. However, whether SILuMAB can simultaneously be used for quantitation and as QC tool for MS-MRD combined with an enrichment step remains to be determined.

Finally, to further evaluate the applicability of MS-MRD, it would be valuable to compare the performance of different MRD detecting methods. However, this would require a large consortium-collaboration. SILuMAB-based MS-MRD does allow upscaling of MS-MRD measurements which makes the performance of such a comparison feasible.

Here, we achieved simplification of MS-MRD for M-protein monitoring by replacing SIL peptides with SILuMAB, an off-the-shelf calibrator. This development will allow for faster, less expensive, and more robust M-protein quantification and will lead to improved harmonization of MS-MRD analysis. Additionally, SILuMAB-based MS-MRD will allow for better QC of the MS-MRD blood-test. We believe this is an important step forwards in the clinical implementation of MS-MRD and associated MS-based techniques.

## Supplementary Material

Supplementary Material

Supplementary Material

Supplementary Material
