# Supplementary material for: M-protein diagnostics in multiple myeloma patients using ultra-sensitive targeted mass spectrometry and an off-the-shelf calibrator
Source: Clin Chem Lab Med. 2023 Oct 12;62(3):540–50. doi: 10.1515/cclm-2023-0781 (PMC10808047; doi:10.1515/cclm-2023-0781)

Supplemental table S1. Patient characteristics

Patient number	Patient ID	PFS in months	M-protein isotype	Follow-up in days	Total sera	M-protein conc. intake sample (g/L)
1	001002	40	IgA-lambda	1268	21	24
2	091006	86+	IgG-kappa	1512	24	64
3	152018	90	IgG-kappa	1407	23	39.1
4	152005	105+	IgG-kappa	1806	30	51.7
5	020001	28	IgA-kappa	909	18	65
6	015005	27	IgG-kappa	894	13	61.4
7	019004	36	IgG-lambda	1092	19	56.3
8	019007	89	IgG-kappa	1693	27	21.1
9	020004	51	IgG-kappa	1491	27	4.9
10	024004	32	IgG-lambda	1063	19	51.7
11	052007	49	IgG-kappa	1567	27	22.5
12	054006	27	IgG-kappa	916	13	68.4
13	091010	92+	IgG-lambda	1418	23	7.1

Supplemental table S2. Peptide sequence of SILuMAB-K1. The five selected SILuMAB peptides are highlighted.

SILuMAB IgG Heavy Chain
EVQLVESGGGLVQPGGSLRLSCVASGFTLNNYDMHWVRQGIGKGLEWVSKIGTAGDRYY
AGSVKGRFTISRENAKDSLYLQMNSLR**VGDAAVYYCAR**GAGRWAPLGAFDIWGQGTMVT
VSSASTK**GPSVFPLAPSSK**STSGGTAALGCLVKDYFPEPVTVSWNSGALTSGVHTFPAVLQ
SSGLYSLSSVVTVPSSSLGTQTYICNVNHKPSNTKVDKKVEPKSCDKTHTCPPCPAPELLG
GPSVFLFPPKPKDTLMISRTPEVTCVVVDVSHEDPEVKFNWYVDGVEVHNAKTKPREEQY
NSTYRVVSVLTVLHQDWLNGKEYKCKVSNK**ALPAPIEK**TISKAKGQPREPQVYTLPPSRDE
LTKNQVSLTCLVKGFYPSDIAVEWESNGQPENNYKTTPPVLDSDGSFFLYSKLTVDKSRWQ
QGNVFSCSVMHEALHNHYTQKSLSLSPG

SILuMAB Kappa Light Chain
QSALTQPRSVSGSPGQSVTISCTGTSSDIGGYNFVSWYQQHPGKAPKLMIYDATKRPSGV
PDRFSGSKSGNTASLTISGLQAEDEADYYCCSYAGDYTPGVVFGGGTKLTVLTVAAPSVFIF
PPSDEQLKSGTASVVCLLNNFYPREAKVQWK**VDNALQSGNSQESVTEQDSK**DSTYSLSST
LTLSKADYEKHKVYACEVTHQGLSSPVTKSFNRGEC

Supplemental table S3. Clonotypic and SILuMAB peptide MS parameters. Clonotypic peptides for which SIL peptides are available are highlighted. (1/2)

	Peptide sequence	M-protein chain	m/z	z	Collision energy (eV)	Fragments
**001002 (patient 1)**	INGDGSVTNYADSVK	IgA-HC	770.3679	2	37.8	y5, y7, y8, y9, y11
	NTLYVEMNGLR	IgA-HC	655.3321	2	33.8	y5, y6, y7, y8, y9
	AIGPVISR	IgA-HC	406.7531	2	26.1	y4, y5, y6, y7, b4
	**YWGQGTLVTVSSASPTSPK**	IgA-HC	983.4995	2	43.5	y5, y6, y8, y10, y12
	**VEDGDEADYYC^{CAM}QVWDSSSAHVVFGGGTR**	Lambda-LC	1036.1088	3	35.9	y5, y6, y18, y19, y22
**091006 (patient 2)**	LSC^{CAM}AGSGFTFSDYGIHWVR	IgG-HC	720.6704	3	38.2	y11, y15, y16, y17, b4
	QAPGNLGLDWVAVISFDGR	IgG-HC	1008.0209	2	52.8	y5, y6, y8, y10, b6
	NEYYGDSVMGR	IgG-HC	645.7746	2	27.7	y5, y6, y7, y8, y9
	**DVDYHGMDVWGQGTTVTVSSASTK**	IgG-HC	1271.0817	2	62.8	y5, y6, y8, y9, y10
	ASQGVTTWMAWYR	Kappa-LC	778.8694	2	31.6	y4, y5, y6, y7, y8
	APQLLIYDASTLEIGVPSR	Kappa-LC	1022.0597	2	53.3	y4, y13, b4, b5, b6
**152018 (patient 3)**	GLEWIGYISYR	IgG-HC	678.8510	2	29.7	y4, y5, y6, y7, y8
	GDTNYNPSLNSR	IgG-HC	669.3077	2	27.1	y6, y7, y8, b5, b6
	VTMSVDTSK	IgG-HC	484.2419	2	22.7	y4, y5, y6, y7, y8
	LSSVTAADTALYYC^{CAM}AR	IgG-HC	881.4274	2	39.7	y5, y6, y8, y9, y10
	**LDHGGNPFMYWGPGTLVAVSSASTK**	IgG-HC	864.7566	3	34.4	y6, y7, y8, y9, y13++
	DIVMTQSPLYLSATPGQPASISC^{CAM}K	Kappa-LC	1282.6404	2	58.5	y7, y10, y11, b4, b7
	FSGSGSVTDFTLEISR*	Kappa-LC	851.9178	2	48.1	y5, y6, y7, y8, y9
**152005 (patient 4)**	LSC^{CAM}AASGFTFSTYSVHWVR	IgG-HC	1088.5177	2	60.4	y4, y6, y7, y9, y11
	**GLEWLAVISGDETTK**	IgG-HC	809.9198	2	32.9	y6, y7, y8, y10, y12
	NTLYLQMNSLRPEDTAVYYC^{CAM}AR	IgG-HC	893.4282	3	34.7	y12, y17, y18, y19, b4
	QAFEDWGLGTLVTVVTVSSASTK	IgG-HC	1198.6208	2	58.9	y6, y8, y9, y11, b8
	YVNWYQQKPGK	Kappa-LC	705.8619	2	40.3	y6, y7, y8, y9, y9++
	**LLIYAASTLENGVPSR**	Kappa-LC	852.4700	2	33.1	y7, y9, y10, y11, y12
**020001 (patient 5)**	**EVQLVESGGALVEPGGSLR**	IgA-HC	949.0049	2	40.0	y4, y5, y6, y7, y8
	NTLYLQMSSLR	IgA-HC	663.3477	2	34.3	y5, y6, y7, y8, y9
	DTQMTQSPSSLSASVGDIITITC^{CAM}R	Kappa-LC	1284.6176	2	60.1	y4, y5, y6, y9, b7
	ASQNIGDFLNWYQQKPGNAPK	Kappa-LC	792.7292	3	36.0	y5, y6, y7, y8, y9
	**LLIFAASNLQSGVPSR**	Kappa-LC	836.9727	2	39.9	y6, y7, y10, y11, y12
**015005 (patient 6)**	**GSGYTFIDYYIHWVK**	IgG-HC	924.9385	2	43.8	y4, y6, y7, y8, y9
	EFQWMGLIDPEDGETR	IgG-HC	961.9205	2	44.6	y7, y8, y9, y11, b4
	EIVLTQSPGILSLSPGER	Kappa-LC	948.5130	2	46.6	y4, y5, y11, y12, b7
	ASQSVPSGYLAWYQQKPGQAPR	Kappa-LC	807.0657	3	46.5	y6, y17, y18, y19, b4
	FSGSGSGTDFALTISR	Kappa-LC	801.8790	2	42.0	y4, y7, y8, y10, y12
	**LEPEDFAVYFC^{CAM}QQYGSSPR**	Kappa-LC	1146.9915	2	59.6	y5, y9, y10, y11, y17
**019004 (patient 7)**	ESGPVLVKPTETLTLTC^{CAM}TVSGFSLSNVR	IgG-HC	998.1753	3	38.3	y6, y8, y9, y10, b16
	**ALEWLAHIFSNDEK**	IgG-HC	836.9075	2	49.7	y5, y6, y7, y8, y9
	DYGLDVWGQGTTVTVSSASTK	Lambda-LC	1086.5135	2	44.0	y6, y12, y14, b5, b6
	**QSVLTQPPSVSAAPGQR**	Lambda-LC	861.9475	2	38.8	y7, y9, y10, y11, y11++
**019007 (patient 8)**	**SDGGTTDYSAPVK**	IgG-HC	649.2860	2	37.6	y4, y5, y6, y10, y11
	NTVYLQMNSLK	IgG-HC	655,8295	2	33.8	y5, y6, y7, y8, y9
	**SSQSLLHSNGNNYLDWYLQKPGQSPQLLIYLGSNR**	Kappa-LC	998.4980	4	40.2	y4, y5, y6, y7, y18
**020004 (patient 9)**	**ASGGIFTNSIITWVR**	IgG-HC	811.4255	2	38.7	y4, y5, y8, y9, y10
	VTITADVSTNTAYMELTSLR	IgG-HC	1093.5415	2	46.2	y5, y6, y7, y8, b6
	GLDYYYYGMDVWGQGTTVTVSSASTK	IgG-HC	1425.1395	2	67.9	y6, y8, b5, b6, b7
	**ASSLEGGVPSR**	Kappa-LC	530.2625	2	24.5	y5, y6, y7,y8, y9
**024004 (patient 10)**	LSC^{CAM}AASGFTFGDYDMHWVR	IgG-HC	1110.4620	2	59.5	y4, y5, y7, y9, b4
	NTLFLQMSDLK	IgG-HC	655.3320	2	30.2	y4, y5, y6, y7, y8
	SGHGDTDFDSWGQGTLVTVSSASTK	IgG-HC	847.3783	3	33.2	y6, y8, y9, b7, b9
	**VEAGDEADYYC^{CAM}QVWDTTTNQGVFGGGTK**	Lambda-LC	1023.4273	3	47.3	y5, y6, y8, y10, y11
**052007 (patient 11)**	LSC^{CAM}AASGFSFSNSAMHWVR	IgG-HC	1057.9570	2	58.9	y4 ,y5, y9, y11, y18
	GLEWVTVISYDGR	IgG-HC	747.8700	**2**	41.0	y5, y6, y7, y8,y9
	**DDSTNTVFLQMNNVR**	IgG-HC	877.3995	2	43.7	y5, y6, y7, y8, y9
	DEDTAMYYC^{CAM}ATSLEYAYDVWGQGAR	IgG-HC	1468.0895	2	63.3	y5, y6, y8, y9, b7

Supplemental table S3. Clonotypic and SILuMAB peptide MS parameters. Clonotypic peptides for which SIL peptides are available are highlighted. (2/2)

	Peptide sequence	M-protein chain	m/z	z	Collision energy (eV)	Fragments
**054006 (patient 12)**	LSC^{CAM}VDSGFTFSDNYMSWIR	IgG-HC	1142.9800	2	55.3	y4, y9, y17, b5, b8
	**QAPGTGLEWIAYISTGSTAIHYADSVK**	IgG-HC	946.1323	3	50.6	y6, y7, y8, y12, y14
	THFGTEQWEVLFDHWGPGTLVTVSSA STK	IgG-HC	805.1353	4	41.1	y7, y8, y9, y6, b7
	**SVSSDLAWYQQK**	Kappa-LC	706.3335	2	34.7	y4, y5, y6, y9, y10
**091010 (patient 13)**	**LSC^{CAM}AAPGFTLNSYAMHWVR**	IgG-HC	727.6657	3	33.8	y6, y9, y14, y15, y16
	GLEWVAVISFDGTNR	IgG-HC	832.4130	2	33.1	y4, y67, y8, y9, y10
	NTLFLQMNSLRPEDTAVYYC^{CAM}AK	IgG-HC	878.7430	3	35.0	y16, y17, y18, y19, y20
	NLNYYYYGIDVWGQGTTVTVSSASTK	IgG-HC	1444.1800	2	63.2	y6, y8, b5, b6, b7
	QSAPTQPASVSGSPGQSITISC^{CAM}TGT SSDVGDYK	Lambda-LC	1090.8223	3	39.1	y4, y20, y23, y22, y27
	**YVSWFQQHPGK**	Lambda-LC	688.8280	2	33.6	y4, y6, y7, y9, y9++
**SILuMAB timsTOF**	EVQLVESGGGLVQPGGSLR	IgG-HC	946.5092	2	40.1	y6, y7, y8, y12, y14
	VGDAAVYYC^{CAM}AR	IgG-HC	627.7942	2	33.6	y4, y5, y6, y7, y10
	GPSVFPLAPSSK	IgG-HC	597.8341	2	32.1	y4, y5, y7, y8, y10
	ALPAPIEK	IgG-HC	423.7624	2	27.2	y4, y4++, y5, y6, y6++
	VDNALQSGNSQESVTEQDSK	Kappa-LC	1072.4951	2	43.6	y4, y5, y6, y8, y9
**SILuMAB orbitrap**	EVQLVESGGGLVQPGGSLR	IgG-HC	946.5092	2	39.9	y6, y12, y13, y14, y15
	VGDAAVYYC^{CAM}AR	IgG-HC	627.7942	2	40.1	y4, y5, y6, y7, y10
	GPSVFPLAPSSK	IgG-HC	597.8341	2	33.6	y4, y5, y7, y8, y10
	FNWYVDGVEVHNAK	IgG-HC	562.6102	3	29.7	Y4, y5, y6, y8, y9
	VYAC^{CAM}EVTHQGLSSPVTK	Kappa-LC	628.6519	3	31.1	y4, y8, y11, y14++, y15++

Supplemental table S4. Additional results for A) linearity and dynamic range, B) variation in longitudinal triplicates C) and correlation between SIL- and SILuMAB quantified MS-MRD data.

**A**	**Linearity and dynamic monitoring**		
	R^2	LoD (g/L)	LLoQ (g/L)
Patient 2	0.994	0.001	0.003
**B**	**CV (%) longitudinal triplicates**		
	No calibrator	SIL	SILuMAB
Patient 4	34	15	14
Patient 5	29	24	18
**C**	**Correlation SIL quantified data - SILuMAB quantified data**		
	R^2		
Patient 4	Erasmus MC 0.998; Radboudumc 0.983		
Patient 5	Erasmus MC 0.998; Radboudumc 0.989		

Supplemental figure S5. Reproducibility comparison between the use of a single M-protein-derived peptide and the use of three M-protein-derived peptides. Three individually digested and measured sera series from patient 1 where the M-protein is quantified by using single M-protein-derived peptides (upper three graphs). The bottom graph shows the M-protein levels observed when the average is calculated over the three M-protein-derived peptides. CVs observed over the three measured series are indicated in yellow, and the average CV over all time points is shown on the right side of each graph.

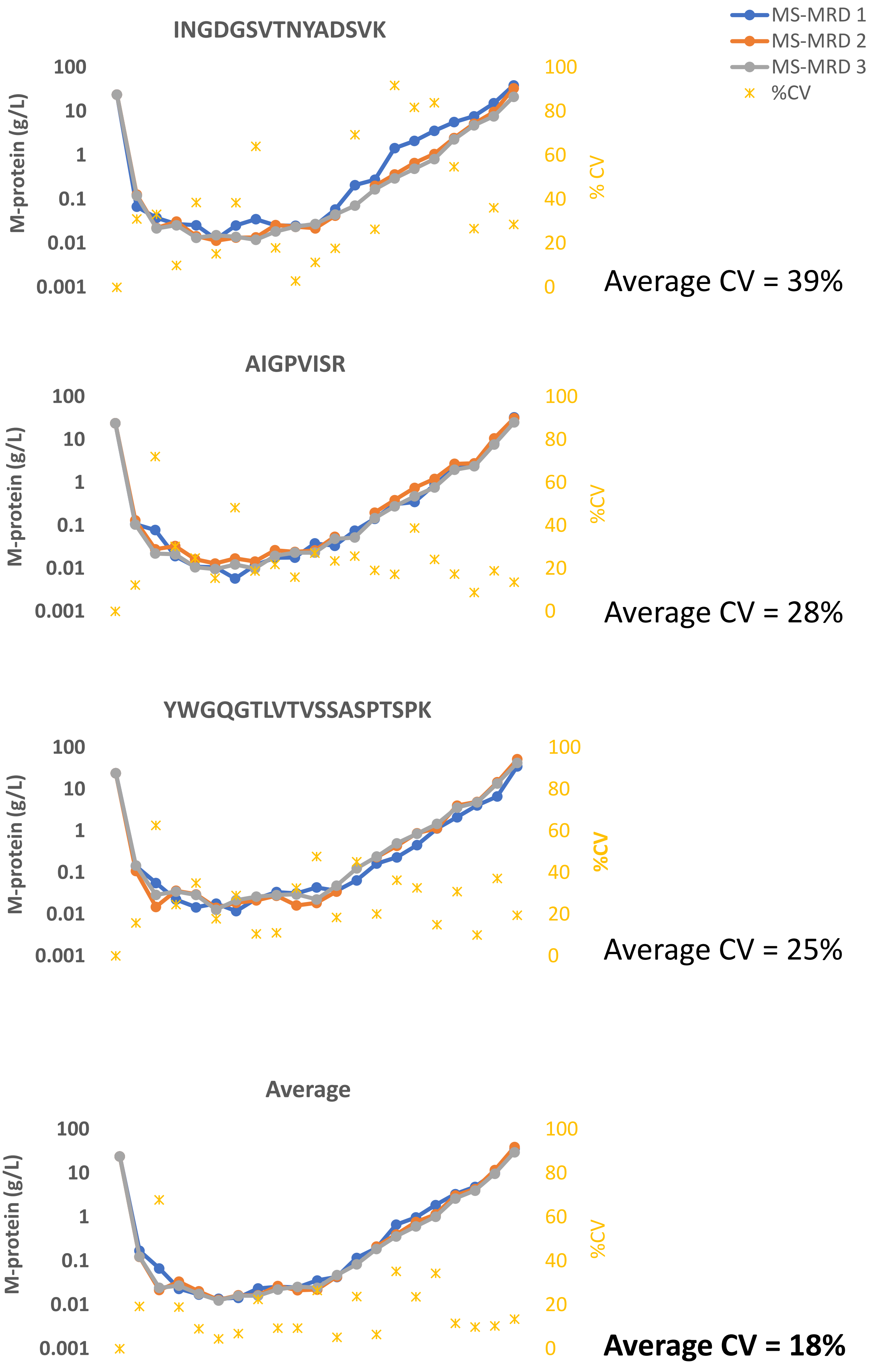

Supplement: Supplementary file 1 — Supplementary Material [file j_cclm-2023-0781_suppl_001.pdf]
