# Supplementary material for: M-protein diagnostics in multiple myeloma patients using ultra-sensitive targeted mass spectrometry and an off-the-shelf calibrator
Source: Clin Chem Lab Med. 2023 Oct 12;62(3):540–50. doi: 10.1515/cclm-2023-0781 (PMC10808047; doi:10.1515/cclm-2023-0781)
Supplement: Supplementary file 2 — Supplementary Material [file j_cclm-2023-0781_suppl_002.docx]

**Supplemental methods to M-protein diagnostics in multiple myeloma patients using ultra-sensitive targeted mass spectrometry and an off-the-shelf calibrator**

**Patient samples**

Sera were collected from the IFM-2009 clinical trial (ClinicalTrials.gov identifier NCT01191060) [1]. Written informed consent and clinical and genomic data were de-identified in accordance with the Declaration of Helsinki, and approval for this study was provided by our institutional review board (2018-4140). The IFM-2009 trial was a phase 3 study that started in 2012 enrolling 700 patients under 66 years who were eligible for autologous stem cell transplantation (ASCT). Induction therapy consisted of three cycles of lenalidomide, bortezomib, and dexamethasone (RVD) followed by either ASCT or five additional RVD cycles. From the 700 patients included in the IFM-2009 trial, we selected patients for this study based on the following inclusion criteria: 1) availability of NGS-MRD data performed pre-maintenance and post-maintenance; 2) availability of RNA sequencing data; and 3) availability of sera at screening, pre-maintenance and post-maintenance [2].

**Clonotypic peptide selection**

The M-protein-specific clonotypic peptide targets were selected based on bioinformatic analysis of a 100,000-read sample extracted from RNA sequencing data as previously described [2, 3]. In short, raw RNA-seq data files were loaded in MiXCR: a software tool specifically designed to analyze T- and B cell receptor sequencing data [4]. The nucleotide sequence of the most abundant clone assembly for both heavy and light chain were translated with IMGT/HIGH-VQUEST software into peptide sequences. Suitable peptides were selected based on the following criteria: length (7-30 amino acids), containing at least one amino acid substitution compared to the germline sequence, as determined by IMGT/HIGH-VQUEST and displaying the highest MS signal intensity.

**Linearity, sensitivity and reproducibility assessment**

To assess linearity and sensitivity of SILuMAB-based MS-MRD, screening serum from patient 1 and 2 were separately serially diluted in control serum. Serial dilutions were prepared from three independent starting concentrations (24 g/L, 12 g/L and 4.8 g/L for patient 1; 64.4 g/L, 32.2g/L and 12.8 g/L for patient 2). All three series were further diluted in 10-fold increments until concentrations of 0.0002, 0.0001, 0.00005 g/L and 0.0006, 0.0003, 0.0001 g/L were reached, respectively. Digestions were performed in independent duplicates that were measured on separate days. Sensitivity was further assessed by testing the limit of blank (LoB), limit of detection (LoD), and lower limit of quantification (LLoQ). LoB was defined as the average M-protein concentration in ten control sera. LoD was defined as LoB + 3.3 * SD_control serum_ and the LLoQ was defined as LoB + 5 * SD_control serum_ as described in the clinical and laboratory standards institute guidelines (https://clsi.org/).

Precision was tested by an intra- and inter-assay variation analysis. To assess the intra-assay variation, four sera from patient 1 containing a high (>5 g/L), medium (0.2 – 0.5 g/L), low (≤ 0.01 g/L), and LLoQ (0.001 – 0.002 g/L) M-protein concentration were analyzed in 20 replicates that were measured consecutively. To test the inter-assay variation, four individual digests of the high, medium, low and LLoQ M-protein samples were measured in five replicates on three different days as recommended by the FDA [5]. Between institute variation was assessed by comparing the results from 13 longitudinally collected patient series (in total 284 unique sera), prepared and measured at two sites using different LC-MS/MS platforms: Radboudumc (EVOSEP one, Odense Denmark – timsTOF pro 2, Bruker Daltonics, Bremen Germany); Erasmus MC (Ultimate - Orbitrap Exploris 120, Thermo Fisher Scientific, Waltham MA U.S.A.)). Henceforth, both sites and platforms will be referred to as timsTOF and orbitrap, respectively.

**Serum digestion**

Serum was diluted 250-fold in MS grade water (Biosolve, Valkenswaard, The Netherlands) containing 7.5% acetonitrile (Biosolve, Valkenswaard, The Netherlands) and 35 mM ammonium bicarbonate (Sigma-Aldrich, St. Louis MO, U.S.A.). To 10 µl of the diluted serum, 1 ng/µl SILu™MAB K1 (Sigma-Aldrich, St. Louis MO, U.S.A.) was added. Proteins were denatured by diluting the samples 1:1 in 0.2 w/v% RapiGest SF Surfactant (Waters, Milford MA, U.S.A.), and 0.7% 0.1 M dithiothreitol (Sigma-Aldrich, St. Louis MO, U.S.A.) was added to reduce cystine bridges. Samples were incubated for 30 minutes at 60 °C in an Eppendorf™ ThermoMixer while mixing at 500 rpm. To alkylate cysteines of the peptides, 0.04% 0.3M iodoacetamide (Sigma-Aldrich, St. Louis MO, U.S.A.) was added, and samples were incubated in the dark for 20 minutes at room temperature. Sequencing grade trypsin (Promega, Madison WI, U.S.A.) was added in a concentration of 70 ng/µg protein and incubated at 37 °C overnight. To stop the digestion reaction, 0.7% Trifluoroacetic acid (Biosolve, Valkenswaard, The Netherlands) was added.

**M-protein quantification by SPEP**

SPEP quantification was performed using a Hydrasys analyzer (Sebia, Envy, France) according to the manufacturer’s protocol. Gel densitometry was recorded, and from these data, M-spikes were calculated in percentages of whole serum protein. Total protein content of the serum was measured using the 2-D quant kit from Cytiva (Marlborough MA, U.S.A.) as per manufacturer’s instructions.

**LC-MS/MS acquisition**

Peptides were separated on a liquid chromatography system (Evosep one). On C18 Evotips (Evosep), 125 ng of digested serum was loaded as per manufacturer’s instructions. Peptides were separated either on a 15 cm (Evosep Endurance, ReproSil-Pur C18, 150 I.D., 1.9 µm particle size) or an 8 cm C18 column (Evosep Endurance, ReproSil-Pur C18, 100 I.D., 3 µm particle size), eluted with a linear gradient of buffer A (HPLC grade water with 0.1% formic acid) and buffer B (acetonitrile with 0.1% formic acid) according to either the Evosep 30 (44 minutes) or the 60 (21 minutes) samples per day method, respectively.

The eluted peptides were analyzed on the timsTOF Pro 2 (Bruker Daltonics, Bremen, Germany) operated in Parallel accumulation-serial fragmentation (PASEF) mode. MS and MS/MS spectra were acquired over 100 to 1700 m/z and the mobility for the TIMS was set from 0.60 to 1.60 Vs/cm^2^. DDA-PASEF analysis was performed to determine the retention time and ion mobility window of peptides. Here, 10 PASEF ramps per 1.17 seconds cycle was used. Intensity threshold was set to 20,000 and active exclusions was set to 0.4 minutes. A 50% duty cycle was used for the TIMS with an accumulation time set to 100 ms and ramp time set to 50 ms. For quantification of the peptides, PRM-PASEF was used. Here, peptide identification results were used by Skyline software to generate a PRM-PASEF method using 5 minutes retention time windows. Clonotypic peptide targets were measured with optimized collision energies. The clonotypic peptides used for quantification are listed in supplemental table S2. Methods used by the Erasmus MC were described previously [6].

**SILuMAB-based MS-MRD data processing**

Data was analyzed as previously described [2]. In short, raw PRM data were loaded into Skyline [7] and the five most intense transitions were selected for both clonotypic and SILuMAB derived peptides (supplemental table S2). A signal was considered positive when the signal to noise ratio was >2 for two or more transitions. Additionally, transition signal compositions were compared to a constructed library from the screening sample by calculating the dot product to measure the degree of similarity. Signals with a dot product < 0.85 were considered negative. For accurate peak fitting an R script was used that integrates peak areas based on log-normal fitting [8]. Subsequently, the peak areas were exported and the M-protein concentrations were determined for each sample using the following formula:

$$\left[ {M protein}_{sample} \right]=\frac{(\frac{Clonotypic peak area in sample}{Average Silumab peak area in sample} )}{(\frac{Clonotypic peak area at intake}{Average silumab peak area at intake})}*\left[ {M protein}_{intake} \right]$$

The M-protein concentration at screening was determined by SPEP. The formula was applied to each clonotypic peptide individually with average SILuMAB peptide intensities for each sample. The concentrations for each clonotypic peptide within a sample were averaged to report one final M-protein concentration.

**Digestion time course data processing**

Raw files were analyzed by PEAKSx+ (version 10.5, BSI). The data was searched against a custom database consisting of the Uniprot (version 2022) database supplemented with the SILuMAB amino acid sequence. The MS1 mass tolerance was set to 15 ppm and the fragment mass tolerance to 0.05 Da with a maximum of 5 missed cleavages. Next to the carbamidomethylation, heavy labelled arginine (6 ^13^C, 4 ^15^N) and lysine (6 ^13^C, 2 ^15^N) were set as fixed modifications and the FDR was set to 1% based on decoy-fusion. Peptide data were exported and analyzed.

**References**

1. Attal M, Lauwers-Cances V, Hulin C, Leleu X, Caillot D, Excoffre M, et al. Lenalidomide, bortezomib, and dexamethasone with transplantation for myeloma. N Engl J Med. 2017 Apr;376(14):1311-20.

2. Langerhorst P, Noori S, Zajec M, de Rijke YB, Gloerich J, van Gool AJ, et al. Multiple myeloma minimal residual disease detection: targeted mass spectrometry in blood vs next-generation sequencing in bone marrow. Clin Chem. Nov 2021;67(12):1689-98.

3. Langerhorst P, Brinkman AB, VanDuijn MM, Wessels HJCT, Groenen PJTA, Joosten I, et al. Clonotypic features of rearranged immunoglobulin genes yield personalized biomarkers for minimal residual disease monitoring in multiple myeloma. Clin Chem. 2021 Jun;67(6):867-75.

4. Bolotin DA, Poslavsky S, Mitrophanov I, Shugay M, Mamedov IZ, Putintseva EV, Chudakov DM, et al. MiXCR: software for comprehensive adaptive immunity profiling. Nat Methods. 2015 May;12(5):380-1.

5. Food and Drug Administration, Bioanalytical method validation guidance for industry. https://www.fda.gov/files/drugs/published/Bioanalytical-Method-Validation-Guidance-for-Industry.pdf (Accessed January 2023).

6. Noori S, Wijnands C, Langerhorst P, Bonifay V, Stingle C, Touzeau C, et al. Dynamic monitoring of myeloma minimal residual disease with targeted mass spectrometry [Letter]. Blood Cancer J. 2023 Feb;13(1):30.

7. MacLean B, Tomazela DM, Shulman N, Chambers M, Finney GL, Frewen B, et al. Skyline: an open source document editor for creating and analyzing targeted proteomics experiments. Bioinformatics. 2010 Apr;26(7):966-8.

8. Stingl C, Luider TM, Applying log-normal peak fitting to parallel reaction monitoring data analysis. J Proteome Res. 2021 Aug;20(8):4186-92.
